# Characterization of mycobacteria and mycobacteriophages isolated from compost at the São Paulo Zoo Park Foundation in Brazil and creation of the new mycobacteriophage Cluster U

**DOI:** 10.1186/s12866-016-0734-3

**Published:** 2016-06-17

**Authors:** James Daltro Lima-Junior, Cristina Viana-Niero, Daniel V. Conde Oliveira, Gabriel Esquitini Machado, Michelle Cristiane da Silva Rabello, Joaquim Martins-Junior, Layla Farage Martins, Luciano Antonio Digiampietri, Aline Maria da Silva, João Carlos Setubal, Daniel A. Russell, Deborah Jacobs-Sera, Welkin H. Pope, Graham F. Hatfull, Sylvia Cardoso Leão

**Affiliations:** Departamento de Microbiologia, Imunologia e Parasitologia, Escola Paulista de Medicina, Universidade Federal de São Paulo, São Paulo, Brazil; Departmento de Ciências Biológicas, Universidade Federal de São Paulo, campus Diadema, São Paulo, Brazil; Centro de Pesquisas Aggeu Magalhães, Fundação Oswaldo Cruz, Recife, Pernambuco Brazil; Departamento de Bioquímica, Instituto de Química, Universidade de São Paulo, São Paulo, Brazil; Escola de Artes, Ciências e Humanidades, Universidade de São Paulo, São Paulo, Brazil; Virginia Bioinformatics Institute, Blacksburg, VA 24060 USA; Department of Biological Sciences, University of Pittsburgh, Pittsburgh, PA 1524 USA

**Keywords:** Mycobacteriophages, *Mycobacterium smegmatis*, Composting, Phage diversity, Whole genome sequencing

## Abstract

**Background:**

A large collection of sequenced mycobacteriophages capable of infecting a single host strain of *Mycobacterium smegmatis* shows considerable genomic diversity with dozens of distinctive types (clusters) and extensive variation within those sharing evident nucleotide sequence similarity. Here we profiled the mycobacterial components of a large composting system at the São Paulo zoo.

**Results:**

We isolated and sequenced eight mycobacteriophages using *Mycobacterium smegmatis* mc^2^155 as a host. None of these eight phages infected any of mycobacterial strains isolated from the same materials. The phage isolates span considerable genomic diversity, including two phages (Barriga, Nhonho) related to Subcluster A1 phages, two Cluster B phages (Pops, Subcluster B1; Godines, Subcluster B2), three Subcluster F1 phages (Florinda, Girafales, and Quico), and Madruga, a relative of phage Patience with which it constitutes the new Cluster U. Interestingly, the two Subcluster A1 phages and the three Subcluster F1 phages have genomic relationships indicating relatively recent evolution within a geographically isolated niche in the composting system.

**Conclusions:**

We predict that composting systems such as those used to obtain these mycobacteriophages will be a rich source for the isolation of additional phages that will expand our view of bacteriophage diversity and evolution.

**Electronic supplementary material:**

The online version of this article (doi:10.1186/s12866-016-0734-3) contains supplementary material, which is available to authorized users.

## Background

Bacteriophages (phages) are the most abundant life forms on the planet, and can influence the entire bacterial ecosystem [1]. They are capable of delivering, through horizontal gene transfer, mechanisms of adaptation and resistance, toxins and photosynthesis genes to their hosts. They also contribute to the cycling of organic and inorganic nutrients [2]. According to the International Committee on Taxonomy of Viruses, double-stranded deoxyribonucleic acid (dsDNA) tailed phages constitute one Order (*Caudovirales*), three Families (*Myoviridae, Podoviridae* and *Siphoviridae*) and 39 Genera (http://www.ictvonline.org/virusTaxonomy.asp?taxnode_id=20120366) [3, 4].

Mycobacteriophages infect members of the genus *Mycobacterium* and were first isolated in the 40’s and 50’s [5, 6]. They were initially used as tools in the study of bacterial genetics and for diagnosis and typing of *Mycobacterium tuberculosis* [7–11]. More than 800 mycobacteriophage genomes have been sequenced (http://phagesdb.org) a collection that has grown rapidly due in part to the impact of integrated research-education programs [12, 13].

All mycobacteriophages isolated to date are dsDNA tailed phages and morphologically are *Siphoviridae* or *Myoviridae* [12]. Mycobacteriophages have been isolated from clinical samples of diseased and healthy people [14, 15], animals [16], laboratory cultures [17, 18], but primarily from the environment, especially from soil to compost samples [19–21]. Most known mycobacteriophages were isolated using *Mycobacterium smegmatis* mc^2^155 as a host, comprising the largest collection of sequenced viruses infecting a common host [22]. However, there is a geographical disequilibrium in mycobacteriophage isolation, and only about 7 % were isolated outside United States of America (USA) (http://phagesdb.org). The analysis of sequenced genomes shows exchange of genome segments among the phage population, revealing mosaicism as the main characteristic of such genomes [22]. Nonetheless, genome comparison allowed the grouping of phages showing significant genomic similarities in clusters and subclusters [23].

Composting is a controlled process of plant and other living material decomposition by autochthone organisms that results in the production of compost to be used as fertilizer. Macro and microorganisms, as common bacteria, fungi, protozoan, algae, larvae and arthropods can participate in the degradation of organic matter. However, composting processes are typically carried out mainly by complex microbial communities [24]. Such intense microbial activity promotes orchestrated changes in temperature and potential of hydrogen (pH) [25]. The high diversity of organisms present at different times during the composting process offers an important source of environmental microorganisms and phages for study.

The São Paulo Zoo Park Foundation (FPZSP) has an area of 825,000 square meters (m^2^) and maintains an exhibition fauna of more than 3,200 animals, as well as the free-living fauna living in the neighboring Atlantic rain forest. The park has operated since 2007 as an environmental management program, including an effluent treatment plant, a water treatment plant and an organic compost production unit, which receives four tons/day of organic residues from Zoo activities and solid waste products from the effluent and water treatment stations. The humus-rich compost generated in the unit is used for the production of food for the Zoo animals in the São Paulo Zoo Farm and for fertilization of the park. The Sao Paulo Zoo composting process has been shown to hold a remarkable bacterial diversity, which can fully account for the biomass degradation [26]. The composting operation was started in 2004 and processes 120 ton/month of organic waste for compost production. Its implementation allowed the recovery and preservation of environmental conditions of the park, reduction of fertilization products costs and better control of the food offered to the Zoo animals. Indirectly, the park obtains financial benefits.

The objectives of this work were to evaluate if compost from São Paulo Zoo Park is a suitable source for isolation of mycobacteria and mycobacteriophages, to understand the dynamics of mycobacterial and phage isolation during the compost processing and to compare isolated phages with mycobacteriophages from other countries. We report the genome sequences of eight newly isolated phages providing insights into viral diversity and the first evidence for mycobacteriophage evolution within a geographically isolated niche.

## Results and discussion

### Isolation and identification of mycobacteria from composting material

Several colonies of acid-fast bacteria were isolated from the São Paulo Zoo Park composting chambers 1, 2 and 3 at each time point (see Table 1), ranging from 2 to 20 per plate (data not shown). This finding is consistent with results obtained in a previous study of metagenomics with two composting chambers from the FPZSP, not related to the ones studied here. The study revealed that Domain Bacteria predominated during the whole process, comprising approximately 90 % of the analyzed sequences and the order *Actinomycetales,* which includes the Family *Mycobacteriaceae*, was included among the 10 most abundant orders [26]. Moreover, Kitamura et al. showed that food waste composts contained relatively high amounts of *Actinobacteria* [27] and Partanen et al. confirmed that Phyllum *Actinobacteria* was predominantly isolated in the thermophilic phase of the composting process [28].

**Table 1:** Cell composition, temperature (mean ± SD) and pH of composting chambers

Date (2011)	chamber 1 vegetables, fruits, manure, beddings and food residues, plant debris and grass clippings, australian goose carcass		chamber 2 vegetables, fruits, manure, beddings and food residues, plant debris and grass clippings, animal carcass and waste water treatment sludge		chamber 3 mixture of partially composted material from chambers 1 and 2		Air T (^o^ C)
	T (^o^ C)	pH	T (^o^ C)	pH	T (^o^ C)	pH	
Mar 11	57,8 ± (16,9)	6,5	55,6 ± (14,7)	6,0			24
Mar 25	56,0 ± (15,7)	7,0	63,4 ± (17,5)	7,5			23
Apr 8	53,0 ± (14,5)	6,0	65,0 ± (18,2)	7,0			23
Apr 28	51,0 ± (13,6)	7,0	55,2 ± (15,7)	7,5			20
May 13					53 ± (15,5)	7,0	23
May 27					62,4 ± (21,5)	7,5	21
June 10					58,2 ± (16,5)	6,5	21

*T* temperature, *° C* degrees Celsius

**Fig. 1:**
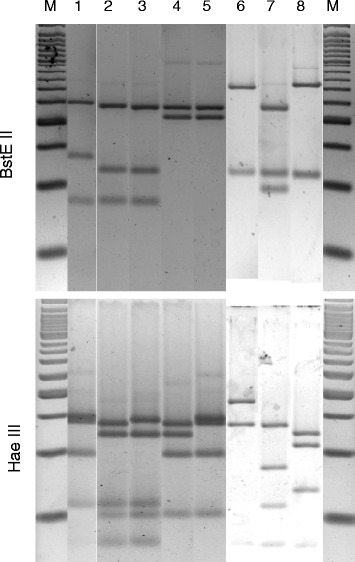
PRA-*hsp65* patterns of isolates recovered from composting materials. BstE II and Hae III restriction patterns of the 441 bp amplicon of the *hsp65* gene are shown in the upper and lower figure, respectively. The figure was produced from different gels with the BioNumerics program v. 7.5 (Applied Maths, Sint-Martens-Latem, Belgium). M: 50 kb ladder (Invitrogen, USA). 1: *M. peregrinum* type 3; 2: *M. fortuitum* type 2; 3: *M. fortuitum* type 1; 4: *M. septicum* type 1; 5: *M. peregrinum* type 1; 6: *M. terrae* type 1; 7: *M. insubricum* type 1; 8: *M. kumamotonense* type 1

Identification of 38 randomly selected isolates from the first two collections by polymerase chain reaction (PCR) restriction enzyme analysis (PRA) of the heat shock protein 65 gene (PRA-*hsp65*) showed that most isolates belonged to the *Mycobacterium fortuitum* group with the following PRA-*hsp65* patterns: *M. fortuitum* 1: BstE II [base pairs (bp)] (235,120,85) and Hae III [bp] (145,120,60,55), *M. fortuitum* 2: BstE II [bp] (235,120,85) and Hae III [bp] (140,120,60,55), *Mycobacterium peregrinum* 1 BstE II [bp] (235,210) and Hae III [bp] (145,140,100,50), *M. peregrinum* 3: BstEI I [bp] (235,130,85) and Hae III [bp] (145,140,100,60) and *Mycobacterium septicum* 1: BstE II [bp] (235,210) and Hae III [bp] (140,125,100,50). Albeit very similar, these PRA-*hsp65* patterns could be clearly distinguished by electrophoresis in 3 % agarose gels (Fig. 1). Other detected PRA-*hsp65* patterns were *Mycobacterium kumamotonense* 1: BstE II [bp] (320,115) Hae III [bp] (130,110,70); *Mycobacterium terrae* 1: BstE II [bp] (320,115) Hae III [bp] (180,130) and a novel profile: BstE II [bp] (235,120,100) Hae III [bp] (140,90,60).

One or two isolates showing each PRA-*hsp65* pattern were submitted to sequencing of the gene that encodes the beta subunit of bacterial ribonucleic acid polymerase (*rpoB*) and of the *hsp65* gene (Table 2). The sequences from isolates showing the PRA-*hsp65* patterns of *M. fortuitum* 1, *M. fortuitum* 2, *M. peregrinum* 1, *M. peregrinum* 3 and *M. septicum* 1 showed highest similarity with sequences from species that belong to the *M. fortuitum* group. This group is composed of *M. fortuitum*, *M. peregrinum*, *Mycobacterium senegalense*, *Mycobacterium conceptionense*, *Mycobacterium alvei*, *Mycobacterium mucogenicum*, *Mycobacterium setense* and the 3^rd^ biovariant sorbitol positive (*Mycobacterium brisbanense, Mycobacterium houstonense* and *Mycobacterium mageritense*) and sorbitol negative (*Mycobacterium bonickei, Mycobacterium neworleansense, Mycobacterium porcinum* and *M. septicum*) [29–32]. The separation of these species is problematic because they share most phenotypic characteristics, the similarity of deoxyribonucleic acid (DNA) sequences is high and the PRA-*hsp65* patterns are very similar, some of them being shared by more than one species: the patterns *M. peregrinum* 2, *M. porcinum* 1 and *M. septicum* 1 are identical, as well as the patterns of *M. senegalense* 1, *M. conceptionenese* 1, *M. houstonense* 1 and *M. neworleansense* 1. A precise definitive identification was only reached for *M. fortuitum* and *M. septicum*, which showed concordant identification results by PRA-*hsp65*, *rpoB* and *hsp65* sequencing. Nevertheless, considering these isolates in conjunct, the *M. fortuitum* group was the most prevalent in compost in this study, corresponding to 76.3 % (29/38) of the obtained isolates.

**Table 2:** Results of identification by PRA-*hsp65,* and by *rpoB* and *hsp65* sequencing of 38 mycobacterial isolates recovered from composting chambers

N	PRA-*hsp65*	*rpoB* ^c^	%	*hsp65* ^c^	%
14	*M. fortuitum* 1^a^	*M. fortuitum* ^T^ (JF346874)	99	*M. fortuitum* ^T^ (AF547833)	100
8	*M. fortuitum* 2^a^	*M. porcinum* ^T^ (AY262737)	98	*M. houstonense* ^T^ (DQ987725)	99
2	*M. peregrinum* 1	*M. septicum* (HM807423)	99	*M. septicum* ^T^ (AF547873)	99
1	*M. peregrinum* 3	*M. alvei* ^T^ (AY859697)	99	*M. peregrinum* ^T^ (AF547865)	99
4	*M. septicum* 1^b^	*M. septicum* (HM807423)	99	*M. septicum* ^T^ (AY496142)	99
5	*M. kumamotonense* 1^a^	*M. kumamotonense* ^T^ (JN571258)	100	*M. kumamotonense* ^T^ (JF491323)	100
3	*M. terrae* 1	*M. senuense* (JN571250)	99	*M. algericum* ^T^ (GU564405)	100
1	novel profile	*M. insubricum* ^T^ (EU022519)	100	*M. insubricum* ^T^ (JF491319)	100

*N* number of isolated colonies analyzed

^a^two isolates from different compost cells were identified by *rpoB* and *hsp65* sequencing

^b^ *M. septicum* 1, *M. peregrinum* 2 and *M. porcinum* 1 share the same PRA-*hsp65* pattern

^c^highest similarity (GenBank accession number)

% similarity ^T^type strain

The isolate showing the PRA-*hsp65* pattern of *M. terrae* 1 was identified as *Mycobacterium senuense* by *rpoB* sequencing and as *Mycobacterium algericum* by *hsp65* sequencing. These species, and *M. kumamotonense,* whose identification was confirmed by *rpoB* and *hsp65* sequencing, belong to the *M. terrae* complex [33, 34], which was the second group most prevalent in compost samples, corresponding to 21 % (8/38) of all isolates.

The isolate showing a novel PRA-*hsp65* pattern not present in the PRASITE database was identified as *Mycobacterium insubricum* by *rpoB* and *hsp65* sequencing. This recently described species was isolated from sputum samples but was not considered clinically significant [35] and from fish [36]. This is the first description of its isolation from environmental samples and PRA-*hsp65* pattern.

### Isolation and purification of mycobacteriophages

Mycobacteriophages were isolated from chambers 1 and 2 using *M. smegmatis* mc^2^155 as host. Crude extracts from the first collection (Mar 11, 2011) yielded 627 lytic plaques with material from chamber 1 and 484 with material from chamber 2. In the second collection (Mar 25, 2011) the number of lytic plaques was reduced to 14 in chamber 1 and 74 in chamber 2. Additional crude extracts collected at intervals of 15 days, were used for phage isolation, but after the fourth collection no lytic plaques were detected, showing that mycobacteriophages able to infect *M. smegmatis* were more prevalent in the initial phases of the process. The same experiment was repeated using two fresh samples from a different chamber, collected at 15-day intervals in the initial phase of the composting process, and 150 and 290 lytic plaques were obtained, respectively. Eight mycobacteriophages randomly selected were purified and stored.

We concluded that compost is a good source of mycobacteriophages capable of infecting *M. smegmatis* mc^2^155. To investigate if mycobacterial isolates from compost could also be used as hosts for isolation of mycobacteriophages from compost we used eight isolates representing the different observed PRA-*hsp65* patterns (*M. fortuitum* 1, *M. fortuitum* 2, *M. peregrinum* 1, *M. peregrinum* 3, *M. septicum* 1, *M. kumamotonense* 1, *M. terrae* 1 and *M. insubricum*) as hosts. Each isolate was infected with the stored material from chambers 1 and 2 and fresh material from two additional chambers. No lytic plaques were obtained in these experiments. Control infections using *M. smegmatis* mc^2^155 as host in the same experiments were successful in generating lytic plaques (data not shown). Moreover, the eight purified phages previously isolated from chambers 1 and 2 were not able to infect the mycobacterial isolates from compost. The inability of phages to infect co-existing strains has been also witnessed in other environments, such as seawater cyanophages [37].

Mycobacteria isolated in this work may not include the natural hosts of mycobacteriophages from compost. Less than 1 % of environmental prokaryotes can grow in controlled conditions [38] and some mycobacteria can be difficult to recover from the environment. Moreover, few known phages infect the mycobacterial species identified in this work. Phage F-phi WJ-1 is the only described mycobacteriophage isolated from *M. fortuitum* [39]*.* Additionally, the protocol used for phage isolation, obtained at http://phagesdb.org/workflow/ included an initial step of filtration of the composting material in 0.22 micrometer (μm) membranes in order to eliminate bacteria and other organisms. This implies that the isolated phages were present as free particles in the material. Occasional phages inside mycobacteria, either free or integrated in the genome, were most probably eliminated in the filtration step. Interestingly, the metagenomic analysis of two composting cells from the FPZSP, distinct from the ones analyzed here, revealed a low amount of viral sequences, corresponding to 0.05 and 0.25 % of the obtained sequences in each chamber [26].

### Genome sequencing

Two sequencing runs yielded 689,550 and 518,987 reads respectively, with 615 nucleaotides (nt) mean length, totaling 424.3 mega base-pairs (Mbp) and 319.4 Mbp, respectively. The reads were assembled generating contigs between 49,117 bp and 69,377 bp. The number of reads in each contig ranged from 2,573 to 161,255. Final assembly revealed that genome sizes varied from 51,355 bp to 69,377 bp. Three phages were shown to have circularly permuted genomes and five had defined termini with 3’ single strand extensions of 10 bases in length (Table 3).

**Table 3:** Characteristics of the sequenced phages. Distribution in clusters and subclusters according to the Mycobacteriophage Database (http://phagesdb.org) and Gene and tRNA prediction performed using the DnaMaster program at http://phagesdb.org/

phage	size (bp)	GC (%)	ORF (#)	tRNA (#)	ends	accession	Sequence of ends (5’–3’)	subcluster
Florinda	59416	61.7	117	0	10-base 3’	KR997930	CGGACGGCGC	F1
Girafales	58456	61.7	112	0	10-base 3’	KR997931	CGGACGGCGC	F1
Quico	58671	61.7	112	0	10-base 3’	KR997968	CCGAAGGCAT	F1
Nhonho	51355	63.8	88	0	10-base 3’	KR997934	CGGATGGTAA	A1
Barriga	52643	63.4	102	0	10-base 3’	KR997929	CGGATGGTAA	A1
Pops	68367	66.6	99	0	Circ perm	KR997967		B1
Godines	67277	69,0	91	0	Circ perm	KR997932		B2
Madruga	69377	50.4	107	1	Circ perm	KR997933		U

The eight phages sequenced here were compared to metagenomic reads obtained from a sample that included material from five different days during the composting process of a single chamber at FPZSP (unpublished work). Some hits were observed, particularly with phage Florinda, in sequences of 583 bp, 460 bp and 352 bp, all with at least 99 % identity. The evidence presented suggests that mycobacteriophage sequences can be at least partially recovered by metagenomics.

By comparison with sequences available in the Mycobacteriophages Database at http://phagesdb.org/ and based on the classification in clusters and subclusters proposed by Hatfull et al. [40], two phages (Nhonho and Barriga) were grouped in Subcluster A1, two phages (Pops and Godines) in Cluster B, one (Pops) from subcluster B1 and the other (Godines) in B2 and three (Florinda, Girafales and Quico) were grouped in Subcluster F1. One phage (Madruga) has high DNA sequence similarity with singleton phage Patience [41], and these two phages were grouped to form the new Cluster U. Functional assignments from database matches, HHPred results, and syntenic placement can be made for 25.6 % of the genes in these genomes. Across the eight genomes, ten new orphams have been identified, four in Subcluster A1 phages Barriga and Nhonho, and six in Subcluster F1 phages, Florinda, Giraffes, and Quico. The genomic features of each of these groups will be discussed in further detail.

### Subcluster A1 mycobacteriophages Nhonho and Barriga

**Fig. 2:**
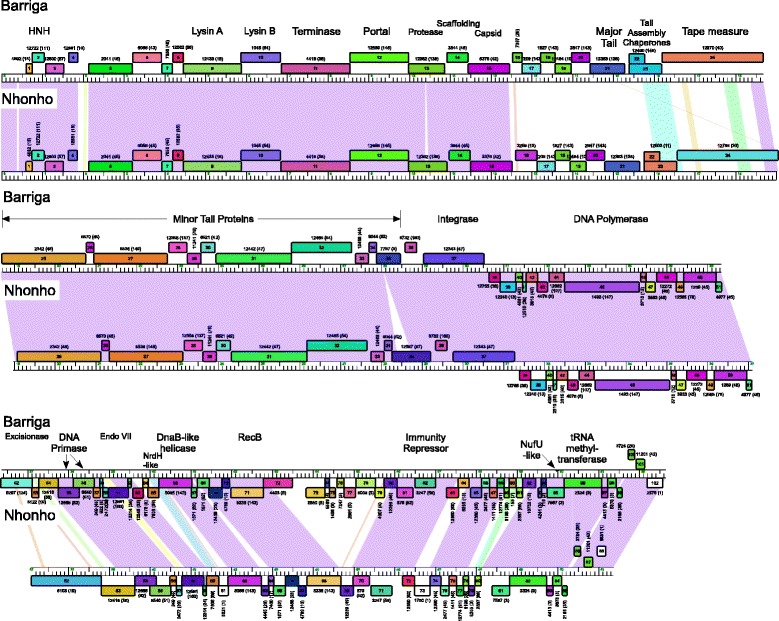
Genome comparisons of Subcluster A1 phages Barriga and Nhonho. Each genome map is shown with the coordinates in kbp flanked by genes represented as boxes positioned above (transcribed rightwards) or below (transcribed leftwards); the phams to which each gene belongs are indicated above or below genes (and colored accordingly) with the number of pham members in parentheses. Pairwise nucleotide sequence similarity between genomes is shown as spectrum-colored shading between the genomes as determined with BLASTN; greatest similarity is shown in purple and weakest similarity (above a threshold level of 10^−4^) shown in red. Barriga and Nhonho have similar gene contents and genome architectures but with a notable insertion/deletion between the *recB* and immunity repressor genes. The nucleotide similarity between the two phages is quite high in the capsid genes in the left arm, disappears almost entirely downstream of the major capsid protein, and reemerges in the minor tail proteins

Phages Nhonho and Barriga belong to Subcluster A1 and share extensive nucleotide sequence similarity across most of their genomes to other A1 phages, including phages from geographically dispersed locations (including China, South Africa and USA) (Fig. 2, Additional file 1: Table S1). Cluster A is the largest of all mycobacteriophage clusters, and includes more than 300 members, almost 40 % of the sequenced mycobacteriophage population. Phages Bxb1 and L5 – members of Subcluster A1 and A2 respectively – were among the first mycobacteriophages to be fully sequenced [42, 43], and Nhonho and Barriga share many genomic features with them (Fig. 2). The structure and assembly genes coding for terminase, portal, protease, scaffolding, capsid, major tail subunit, tail assembly chaperones, tape measure and minor tail proteins have the canonical syntenic organization, and the lysis cassette is located between the structural genes and the genome left end. The integration cassette that includes a serine-integrase is positioned near the center of the genome and separates the rightwards-transcribed structural genes of the left arm from the leftwards-transcribed genes in the right arm. The repressor genes (Nhonho *71* and Barriga *82*) are closely related to the Bxb1 gp69 repressor (98 and 100 % aa identity respectively) and presumably are homoimmune to Bxb1 [44] and the other Subcluster A1 phages.

Although Nhonho and Barriga have similar genomic architectures, there are several informative differences between them (Fig. 2). For example, in the Barriga right arm there are eight open reading frames of unknown function (*72* – *79*) that are absent from Nhonho, but present in a small subset of other Subcluster A1 phages. We also note that Barriga *84* is replaced by gene *73* in Nhonho, an orphan with no close relatives in the mycobacteriophage database. Finally, we note that Barriga gp52, which is a homologue of the Bxb1 Recombination Directionality Factor (RDF) gp47 [45] lacks an intein that is present in the homologue (gene *52*) in Nhonho.

**Fig. 3:**
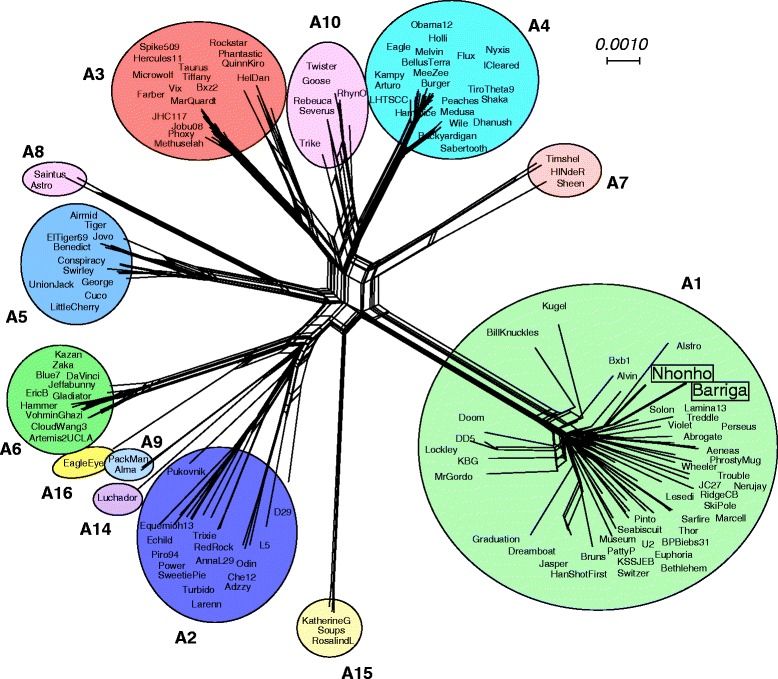
Gene content-based network relationships among Subcluster A mycobacteriophages. Splitstree [59] was used to present the relationships between Cluster A mycobacteriophages, using the Phamerator database ‘Actinobacteriophage_554’, filtering for taxa within Cluster A. Colored circles indicate the different subclusters, and the phages isolated from the Sao Paulo zoo are boxed

Comparison of the gene contents of Barriga and Nhonho with those of other Cluster A phages illustrates that these two phages are more closely related to other Subcluster A1 genomes than those in other subclusters within Cluster A (Fig. 3). The Subcluster A1 phages are numerous and diverse, but Barriga and Nhonho are nonetheless quite closely related to each other, suggesting the possibility that they have evolved together relatively recently within the geographical region from which they were isolated.

### Mycobacteriophages Pops and Godines from Cluster B

**Fig. 4:**
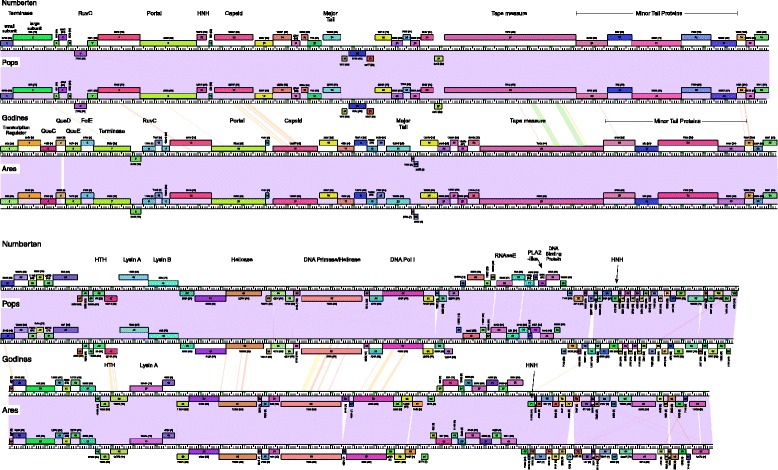
Genome comparisons of Cluster B phages Numberten, Pops, Godines and Ares. Phages Numberten and Pops are highly similar in both nucleotide sequence and gene content across entire genome spans, as are Godines and Ares; see Fig. 2 for figure annotation details. All four Cluster B phages have similar gene contents and genome architectures, with insertion/deletions reflected in interruptions in the purple shaded regions between genome pairs

Phages Pops and Godines belong to Subclusters B1 and B2, respectively, with close similarity to phages isolated elsewhere, notably within the USA (Additional file 1: Table S1) [46]. Pops is most closely related to the Subcluster B1 phages Numberten and Suffolk, but differs from both in several genomic features (Fig. 4). Several of these differences suggest genes that are unlikely to be essential for lytic growth as they are present in some but not all of these genomes: First, Pops and Numberten share a gene (gene *54*) of unknown function that is absent in Suffolk; second, Pops lacks a small gene (50 codons) present in both Suffolk and Numberten (genes *61* and *62* respectively), third, Pops *79* and Suffolk *78* are nearly identical, but the corresponding gene in Numberten (*80*) has a central deletion removing about 50 % of the gene (Fig. 4). Finally, Pops gene *92* is closely related to Numberten gene *95*, but is deleted in Suffolk.

The Subcluster B2 phage Godines is most closely related to phages Ares and Arbiter (Fig. 4). Godines shares the common Subcluster B2 features such as the lack of Lysin B gene [47–49]. Another characteristic of this group is the presence of six genes between the gene for terminase and the left genome end (Fig. 4), whose products are predicted to be involved in the synthesis of queuosine from guanosine-5’-triphosphate (GTP).

Informative differences between Godines and other B2 genomes include the insertion of a small gene (*56*) of unknown function between the putative primase/helicase and DNA polymerase genes, which is has only one other relative present in the mycobacteriophages, in the Subcluster B5 phage Baee (Fig. 4). Also, a gene present in both Ares and Arbiter (*77* and *74* respectively) is absent from Godines, and a Godines gene (*89*) is present in Ares (gene *90*) but is absent from Arbiter; presumably these genes are not required for lytic growth.

Pops and Godines share only low DNA sequence similarly to each other and share fewer than 25 % of their genes through amino acid sequence comparisons. They are each more closely related to other phages than to each and thus there is no evidence to suggest they have evolved in evolutionary isolation in the geographic area from where they were isolated.

### Cluster F mycobacteriophages Florinda, Girafales and Quico

**Fig. 5:**
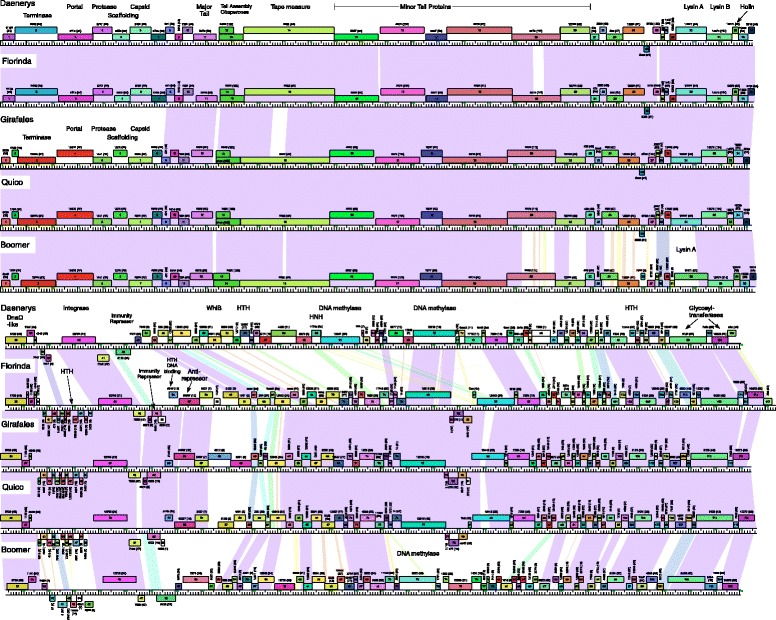
Genome comparisons of Cluster F mycobacteriophages Florinda, Girafales, Quico, Danaerys, and Boomer. Florinda has close similarity with Danaerys capsid and packaging genes near the left end; Girafales and Quico have greatest similarity in this region to Boomer. All three newly sequenced phages have a region containing 12 genes in common adjacent to the integrase that are either not present or rare in other Cluster F phages

**Fig. 6:**
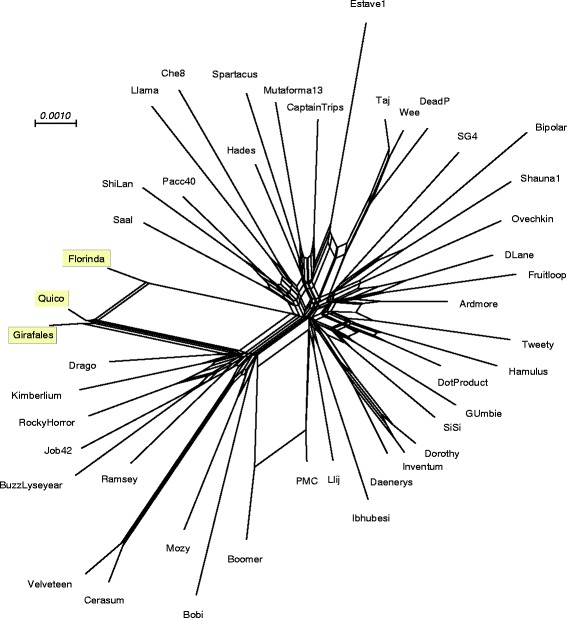
Gene content-based network relationships among Subcluster F1 mycobacteriophages. Splitstree [59] was used to present the relationships between Subcluster F1 phages, using the Phamerator database ‘Actinobacteriophage_554’, filtering for taxa within Subcluster F1. Phages isolated from the Sao Paulo zoo are highlighted in yellow

Three phages, Florinda, Girafales, and Quico are related to Cluster F phages, and all belong in Subcluster F1 (Fig. 5). Nucleotide similarity searches using Nucleotide Basic Local Alignment Search Tool (BLASTN) suggest that the three phages are somewhat more similar to each other than to other Subcluster F1 phages, and this is reflected in a phylogenetic representation based on gene content (Fig. 6). This presents compelling evidence that these three phages have evolved with some degree of isolation from the other Subcluster F1 phages, most of which were isolated within the United States, as suggested for Nhonho and Barriga above. Interestingly, there is no close general correlation between geological location and genome type for the mycobacteriophage phage collection as a whole, and the relatedness between Florinda, Girafales and Quico may indicate localization within the specific zoo location from which they were isolated. Comparison of the genome maps (Fig. 5) shows that the gene content relationships are likely dominated by a series of 12 small open reading frames (29–86 codons; Girafales genes *38*–*50* and their homologues) located immediately to the left of the integrase gene. These small open reading frames (ORFs) are nearly identical in Florinda, Girfales and Quico but not found in any other mycobacteriophage genome (Fig. 5).

Other notable features of these phages include the 7–8 genes – including the terminase, portal, and capsid subunit genes – at the extreme left end of the genomes (Fig. 5). The Cluster F genomes have two distinct types of these genes that have unrelated DNA sequences but encode similar functions: Florinda has one distinct type (related to genes in phages such as Daenerys and Llij), whereas Girafales and Quico have the alternative type (related to phages Boomer and Ramsey). Secondly, there is a small group of genes in Girafales (*58, 59, 62*) that substitute for unrelated genes in Florinda and Quico (Fig. 5). We also note that a small gene (*115*, 55 codons) present at the right end of the Florinda genome is absent from both Quico and Girafales, although found in otherwise unrelated genomes in Subclusters A1, D1 and D2. Its function is unknown (Fig. 5).

### Mycobacteriophage Madruga and the novel Cluster U

**Fig. 7:**
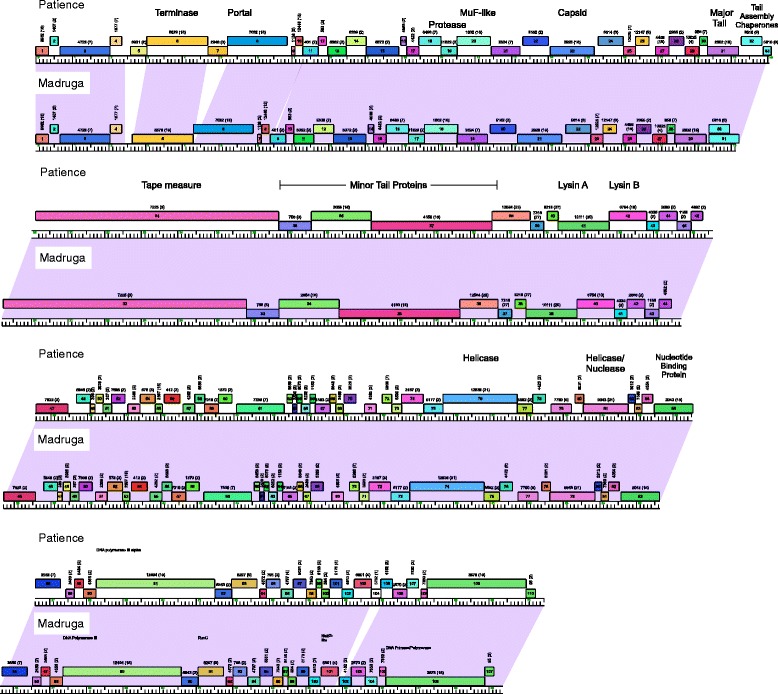
Genome comparisons of Cluster U phages Madruga and Patience. Phages Madruga and Patience share extensive nucleotide sequence similarity with three notable insertion/deletions, and constitute the new Cluster U; see Fig. 2 for figure annotation details

Phage Madruga is very closely related to the singleton mycobacteriophage Patience, with similarity spanning >95 % of their genome lengths (Table 3) [41], and as such, these two phages constitute a newly assigned Cluster U (Fig. 7). Patience has an unusually low percent of guanine and cytosine (GC%) content (50.3 %) and its proteome has been defined by mass spectrometry; it was isolated in 2009 in Durban, South Africa [41]. Madruga therefore offers new perspectives from the comparative genomic analysis.

First, Madruga shares the low GC% content (50.4 %) to that of Patience, and the genomes are of similar lengths (69,377 bp and 70,506 bp in Madruga and Patience respectively). The length difference is primarily accounted for by three genes that are present in Patience but absent from Madruga: Patience genes *5, 7* and *104* (Fig. 7)*.* Patience *5* is a putative histidine-asparagine-histidine motif (HNH) endonuclease and there are distantly related copies in Cluster C phages Myrna and Dandelion (39 % and 33 % aa identity respectively); the protein is expressed both early and late in lytic growth [41]. Patience *7* is related to the Holliday Junction resolvase endonuclease VII which is expressed late in lytic growth [41] and although this function is likely required for DNA packaging, we note that Patience and Madruga both encode another Holliday Junction resolvase (RusA-like; genes *91* and *93* respectively) elsewhere in the genome that is expressed late in lytic growth and could provide functional redundancy (Fig. 7). Finally, Patience gene *104* is absent from Madruga and was shown to be expressed late in Patience lytic growth but is presumably not required; its function is unknown.

Finally, we note that putative tails proteins in both Madruga and Patience (gp34 and gp36 respectively) contain a C-terminal 150-residue domain that is also present at the C-termini of putative tail proteins encoded by other phages, including Pops (and other Subcluster B1 phages), and phages in Subcluster D1, D2, H1, H2, and Cluster R (Fig. 7). HHPred analysis strongly indicates that this domain has a carbohydrate binding function, perhaps with Endo-1,4-beta-xylanase activity. Presumably this is associated with host surface recognition or capsule degradation to facilitate the early stages of infection.

## Conclusions

In conclusion, this work provides new insights into the genetic diversity of mycobacteriophages, and presents supporting evidence for local evolution of groups of phages (Florinda, Quico and Girafales; Barriga and Nhonho) in relative isolation from other mycobacteriophages. Although the database of sequenced mycobacteriophage genomes is quite large, this is the first clear evidence for this phenomenon, and may reflect in part the richness and growth of the microbial communities within the zoo composting systems from which they were isolated. Interestingly, none of the phages isolated here appear to infect any of the mycobacterial strains isolated from the same locations, and thus the natural host for these phages is unclear. We predict that composting systems such as those used to obtain these mycobacteriophages will be a rich source for the isolation of additional phages that will expand our view of bacteriophage diversity and evolution.

## Methods

### Composting chambers

Composting material from 8 cubic meters (m^3^) chambers was collected for this work. Two chambers (1 and 2) were followed and four collections were done from March 11 to April 28, 2011. After this period the partially composted material from both cells was reunited in a third chamber, which was followed until June 10, with three additional collections. This is the regular procedure of compost production carried out at the FPZSP. At each collection, the temperature of five points located 1.5 meters (m) bellow the cell surface was checked and the mean temperature of the five points was calculated. The pH of the collected material was measured using a pH measure strip (EM Science, Germany). The composition, mean temperature and pH of each chamber and the air temperature at each collection are shown in Table 1. At each time point, material from five different points from each chamber was collected and pooled as previously described [50]. The material was aliquoted in two 50 mililitre (mL) tubes, one used for mycobacteria and mycobacteriophage isolation and the other for storage at −20 °C.

### Bacterial strains

*M. smegmatis* mc^2^155 was cultivated on solid Middlebrook 7H10 medium (Becton Dickinson and Company, Franklin Lakes, NJ, USA) supplemented with 10 % OADC (oleic acid, albumin, dextrose and catalase - Becton Dickinson and Co). Isolated colonies were then transferred to liquid Middlebrook 7H9 medium (Becton Dickinson and Co) supplemented with 10 % OADC, Tween 80® 0.05 % and CaCl_2_ 1 mM (Merck, Darmstadt, HE, Germany) and cultivated at 37 °C for 24 h. The culture was diluted 1:20 in the same medium without Tween 80 until 0.5 < D.O._600nanometers(nm)_ < 0.7 (log phase) was reached.

### Isolation of mycobacteria from compost

Isolation of mycobacteria was carried out as described [51]. The collected material [10 gram (g)] was diluted in 40 mL of sterile water and maintained in a shaker for 1 h (h) at room temperature (RT). The solution was centrifuged at 600 force of gravity (*g*) for 5 min (min) at 4 ° C for precipitation of large particles and the supernatant was transferred to sterile tubes and centrifuged at 8000 *g* for 15 min at 4 ° C. Sodium dodecyl sulfate (SDS) 3 %/NaOH 4 % were added to the pellet, which was incubated for 15 min at RT. The sample was centrifuged again under the same conditions and the pellet was treated with a solution of cetrimide (Henrifarma Produtos Químicos e Farmacêuticos, São Paulo, Brazil) 2 % for 5 min. After centrifugation under the same conditions, the pellet was washed and resuspended in 0.5 mL of deionized water. An aliquot of 0.1 mL was distributed in duplicates on Löwenstein-Jensen (Probac do Brasil, São Paulo, Brazil) slants and on 7H10-OADC plates supplemented with PANTA (antibiotic solution containing final concentrations of 40 U/mL polymyxin, 4 μg/mL amphotericin B, 16 μg/mL nalidixic acid, 4 μg/mL trimethoprim and 4 μg/mL azlocillin) [52]. The cultures were incubated at 30 ° C and 37 ° C and examined every 2 days in the first 10 days and once a week for a total of 90 days.

### Identification of mycobacteria

Isolated colonies were visualized by microscopy after Ziehl-Neelsen staining. Acid-fast bacilli were identified by PRA-*hsp65*. DNA was extracted by boiling a loop full of acid-fast bacilli taken from solid plates in 300 microlitres (μL) of TET [Tris–HCl 10 milimolar (mM), EDTA 1 mM, pH 8.0, Tween 20® 0.05 %] for 10 min. A 441 bp fragment from the *hsp65* gene was amplified using primers Tb11 (5’-ACCAACGATGGTGTGTCCAT-3') and Tb12 (5’-CTTGTCGAACCGCATACCCT-3') as previously described [53]. Amplified products were separately digested with BstE II at 60 ° C and Hae III at 37 ° C for 90 min. Digestion products were separated by electrophoresis in 3 % agarose gels stained with ethidium bromide and the resulting bands were analyzed by comparison with restriction profiles at the PRASITE (http://app.chuv.ch/prasite/index.html).

The identification of 11 selected isolates was confirmed by DNA sequencing. Partial sequences of *rpoB* and *hsp65* genes were obtained. The primers used for amplification and sequencing of *rpoB* were MycoF (5'-GGCAAGGTCACCCCGAAGGG-3') and MycoR (5'-AGCGGCTGCTGGGTGATCATC-3') [54] and for *hsp65* were hsp667-forward (5'-GGCCAAGACAATTGCGTACG-3') and hsp667-reverse (5'-GGAGCTGACCAGCAGGATG-3') [55]. The amplicons were purified using QIAquick PCR purification Kit (Qiagen, Germany). Dideoxy sequencing was performed using BigDye® Terminator v 3.1 Cycle Sequencing Kit (Applied Biosystems, USA) and run in ABI PRISM 3100 DNA Analyzer (Applied Biosystems). The sequences were analysed by comparison with sequences deposited in the GenBank using the Basic Local Alignment Search Tool (BLAST: http://blast.ncbi.nlm.nih.gov/Blast.cgi).

### Mycobacteriophage isolation

Approximately 30 g of collected material were diluted 1:1 weight/volume percent (w/v) in phage buffer (Tris 10 mM pH 7.5, MgSO_4_ 10 mM, NaCl 68 mM, CaCl_2_ 0.1 mM). After homogenization and incubation at RT for 30 min, the samples were centrifuged at 3,500 *g* for 15 min (Sorvall, Newtown, CT, USA) and the liquid phase was filtered through a 0.22 μm pore membrane (Techno Plastic Products AG-TPP, Trasadingen, SH, Switzerland). A volume of 10 μL of the filtered material was incubated with 0.5 mL of log-phase cultures of *M. smegmatis* mc^2^155 or mycobacterial isolates from compost at RT for 30 min and added to 2.5 mL of Middlebrook top agar (MBTA)/CaCl_2_ (7H9 liquid medium with agar 0.7 % and CaCl_2_ 0.1 mM) and 2 mL of 7H9-OADC. The final volume was homogenized and poured over solid 7H10-OADC/CaCl_2_ plates, which were incubated for 16 h at 37 ° C. Negative controls (no addition of compost filtered material) and positive controls (phage D29) were included in each experiment. After the incubation period, putative lytic plaques showing different sizes and morphologies were collected by removing the agar regions using micropipet tips and transferred to microcentrifuge tubes containing 100 μL of phage buffer. Several rounds of purification were performed by infection of *M. smegmatis* mc^2^155 until all lytic plaques in each sample showed the same size and morphology. Each purified phage was then amplified and titered. The amplified phage samples were harvested, filtered through a 0.22 μm membrane and ultracentrifuged at 65,000 *g* at 4 °C for 2 h (Beckman-Coulter Optima XL 100 K, Pasadena, CA, US). The pellet was suspended in 1 mL of ammonium acetate 0.1 molar (M) (Synth, Brazil), pH 7.5 or in phage buffer.

### Whole genome sequencing (WGS), assembly and annotation of phages genomes

DNA from purified phages was submitted to pyrosequencing using standard Roche 454 GS FLX Titanium protocols (Roche Applied Science), at the Center for Advanced Technologies in Genomics (CATG), Instituto de Quimica, Universidade de Sao Paulo. Barcoded (tagged) shotgun libraries for each DNA sample were constructed using GS Titanium Rapid Library Prep Kit, pooled and submitted to two sequencing runs. Sequencing reads were separated according to their individual tags, quality-filtered and individually assembled using 454 Newbler assembler software version 2.5.3. Automatic annotation was performed using DNA Master software v. 5.0.2 (JG Lawrence, http://cobamide2.bio.pitt.edu/computer.htm), followed by manual curation. Hypothetical protein genes were analyzed for the presence of conserved domains using online tools CDD-NCBI [56] and InterPro-EMBL-EBI [57]. Genome maps were generated using Phamerator [58] with the database ‘Actinobacteriophage_554’. This database contains 554 phage genomes of phages infecting Actinobacterial hosts, consisting of 56,572 genes, organized into a total of 8032 phamilies (phams) composed of sequence-related proteins. Parameters for pham construction were as described previously [22]. Network relationships were derived from the Phamerator Pham table, and represented using Splitstree selecting only the Cluster F1 phages.

## Abbreviations

BLAST, basic local alignment search tool; BLASTN, nucleotide basic local alignment search tool; bp, base-pair; CATG, center for advanced technologies in genomics; DNA, deoxyribonucleic acid; dsDNA, double-stranded deoxyribonucleic acid; FPZSP, Sao Paulo Zoo Park Foundation; *g*, force of gravity; g, gram; GC%, percent of guanine and cytosine; GTP, guanosine-5’-triphosphate; h, hour; HNH, histidine-asparagine-histidine motif; PCR, polymerase chain reaction; *hsp65*, heat shock protein 65 gene; m, meter; M, molar; *M.*, *Mycobacterium*; m^2^, square meter; m^3^, cubic meter; Mbp, mega base-pair; MBTA, Middlebrook top agar; min, minute; mL, mililitre; mM, milimolar; nm, nanometer; nt, nucleotide; OADC, oleic acid, albumin, catalase and dextrose supplement; ORF, open reading frame; PANTA, antibiotic solution containing final concentrations of 40 U/mL polymyxin, 4 μg/mL amphotericin B, 16 μg/mL nalidixic acid, 4 μg/mL trimethoprim and 4 μg/mL azlocillin; PCR, polymerase chain reaction; pH, potential of hydrogen; PRA-*hsp65*, polymerase chain reaction restriction enzyme analysis of the *hsp65* gene; *rpoB*, gene that encodes the beta subunit of bacterial ribonucleic acid polymerase; RT, room temperature; SDS, sodium dodecyl sulfate; USA, United States of America; w/v, weight/volume percent; WGS, whole genome sequencing; μL, microlitre; μm, micrometer

## Additional file

Additional file 1: Table S1. Similarity of sequenced phages with phages deposited in the GenBank. The phage genomes sequences were compared with sequences deposited in the GenBank using the Basic Local Alignment Search Tool (BLAST: http://blast.ncbi.nlm.nih.gov/Blast.cgi). (XLSX 49 kb)
